# Advancements in Vaccine Strategies for Chronic Liver Disease Patients: Navigating Post-COVID Challenges and Opportunities

**DOI:** 10.3390/vaccines12020197

**Published:** 2024-02-15

**Authors:** Samer Al-Dury, Nelly Kanberg

**Affiliations:** 1 Department of Medicine, Gastroenterology and Hepatology Unit, Sahlgrenska University Hospital, 413 45 Gothenburg, Sweden; 2 Institution of Medicine, University of Gothenburg, 405 30 Gothenburg, Sweden; 3 Department of Infectious Diseases, Sahlgrenska University Hospital, 416 85 Gothenburg, Sweden; nelly.kanberg@gu.se; 4 Institution of Biomedicine, University of Gothenburg, 405 30 Gothenburg, Sweden

**Keywords:** cirrhosis, chronic liver disease, vaccination recommendations, vaccine response, vaccine technology

## Abstract

This review addresses the vital role of vaccinations in managing patients with chronic liver disease (CLD), especially in the context of the post-COVID-19 landscape. The pandemic has highlighted the unique vulnerabilities of CLD patients, including those awaiting liver transplantation and post-transplant individuals, who face heightened risks of infection due to compromised immune responses. Recent advancements in vaccine technology, such as mRNA platforms, novel adjuvants, and advanced delivery systems, have significantly accelerated vaccine development, enhancing both speed and efficacy. Moreover, the emergence of personalized vaccines, tailored to everyone’s unique immunological profile, presents new opportunities, particularly for those with chronic conditions. This review synthesizes the current state of evidence regarding vaccine recommendations for CLD patients, focusing on their response to vaccinations and proposing effective strategies to protect this vulnerable group from vaccine-preventable diseases. It also explores the challenges in implementing these strategies and considers the impact of emerging vaccine delivery systems on improving outcomes for CLD patients. The paper aims to provide nuanced guidance on vaccination in the rapidly evolving healthcare landscape, addressing both technological innovations and comprehensive patient care strategies.

## 1. Introduction

Chronic liver disease (CLD) represents a significant global health issue, impacting a vast demographic across the globe due to its multifactorial etiologies such as alcohol use disorder (AUD), metabolic dysfunction-associated steatotic liver disease (MASLD), viral hepatitis, and autoimmune disorders [1]. These underlying conditions severely impair the immune system, thereby elevating the susceptibility of CLD patients to infectious diseases and complicating their clinical management. The advent of the COVID-19 pandemic has further accentuated these vulnerabilities, particularly in individuals with advanced CLD or liver cirrhosis, by exposing them to an escalated risk of severe infections and an associated increase in mortality rates [2].

The post-COVID era has underscored the critical necessity for robust and effective vaccination strategies tailored to the unique immunological profile of CLD patients. The hallmark of the immunological distortion in CLD is collectively known as cirrhosis-associated immune dysfunction (CAID) [3]. A crucial aspect of CAID is diminished vaccination response. This encompasses a range of immune anomalies such as dysregulated cytokine production, altered leukocyte populations, and impaired functions of antigen-presenting cells. This dysregulation severely limits the body’s innate and adaptive immunity, undermining its ability to combat pathogens and respond adequately to vaccinations [4,5].

Amidst these challenges, vaccine hesitancy remains a formidable barrier to achieving comprehensive immunization coverage within this patient group. Factors contributing to this hesitancy include socioeconomic disparities, fears regarding adverse effects, and skepticism about vaccine efficacy, necessitating targeted communication strategies and evidence-based interventions to mitigate these concerns [6]. Despite the general advocacy for vaccinations against influenza, pneumococcal disease, and viral hepatitis among CLD patients, there is a notable deficiency in systematic evaluations of vaccine efficacy specifically within this demographic with much of the supporting evidence extrapolated from studies on other immunocompromised populations [7].

Recent advancements in vaccine technology, notably the emergence of mRNA vaccine platforms, offer promising avenues for enhancing vaccine efficacy, compliance, and safety in the broader population. However, the specific immunological challenges presented by CLD patients have not been the primary focus of these developments, and patients with cirrhosis were excluded from the clinical trials evaluating mRNA vaccines efficacy and safety, often relegating the assessment of immune responses in such compromised groups to a secondary consideration [8,9].

The objective of this review is to provide clear and nuanced guidance on the current vaccine recommendations for patients with CLD with and without cirrhosis. Recognizing the diversity in recommendations across various medical societies, this review seeks to synthesize the current state of evidence, focusing on how CLD patients respond to vaccinations. We aim to propose effective strategies for protecting this vulnerable group from vaccine-preventable diseases. Additionally, the review will briefly explore the challenges in implementing these strategies and consider the potential impact of emerging vaccine delivery systems on improving outcomes for CLD patients.

## 2. Materials and Method

We conducted a detailed examination of vaccination guidelines from authoritative health organizations, including the Centers for Disease Control and Prevention (CDC), the American Association for the Study of Liver Diseases (AASLD), the European Association for the Study of the Liver (EASL), and additional national societies. Our methodology consisted of an analysis and synthesis of these guidelines to develop coherent and comprehensive vaccination recommendations for patients with CLD with and without cirrhosis.

To ensure the relevance and precision of our research, we employed specific inclusion and exclusion criteria. We assessed studies focused on vaccination responses exclusively in CLD patients and those on the liver transplant waiting list. Excluded from our review were studies addressing acute liver failure, significant decompensation events, or acute-on-chronic liver disease scenarios, as well as studies including patients with malignancies or those receiving high-dose immunosuppressive therapy, which could confound vaccine response data.

## 3. Results

The recommendations formulated in this narrative review are intended to accurately represent the typical CLD patient demographic encountered in community healthcare environments across various disease stages. We evaluated vaccine formulations and administration protocols, taking into account dosage, number of doses, and dosing intervals. Vaccine formulations and the method of administration follow standard practice for the majority of immunizations in healthy populations. Alternative delivery methods and tailored booster doses are briefly mentioned whenever they are clinically relevant. Our analysis, while comprehensive, is tailored to be particularly relevant for the majority of CLD patients, aiming to improve adherence and pragmatic application in day-to-day clinical practice.

### 3.1. Hepatitis A and B viruses (HAV and HBV)

Several international societies recommend hepatitis A vaccination for all patients with CLD. Studies show that while 95% of these patients seroconvert after receiving the two-dose inactivated vaccine, this rate is slightly lower than the 98% observed in healthy individuals [10]. Moreover, hepatitis A vaccine-generated antibody titers tend to be lower in patients with liver disease, especially when cirrhosis is present. This pattern has also been observed in those with hepatitis B [11] and in children with CLD [12].

Patients with advanced or decompensated liver disease have even lower vaccine effectiveness with seroconversion rates ranging from 49% to 66% [13,14,15]. In liver transplant recipients, vaccine immunogenicity is further compromised, and antibody titers may decline over time [16,17].

In terms of safety, the inactivated hepatitis A vaccine is well tolerated in patients with CLD, presenting minimal side effects and requiring no special post-vaccination precautions. Although its effectiveness is reduced in immunocompromised individuals, no serious adverse events have been associated with the vaccine. Currently, we do not recommend post-vaccination monitoring of anti-HAV antibodies.

Hepatitis B vaccination is strongly recommended for all patients with CLD [18,19]. Despite this recommendation, the actual vaccination coverage is alarmingly low. In the United States, only about 30% of CLD patients have received the hepatitis B vaccine, fewer than 30% completing the series [20]. In Sweden, the practice of screening and vaccinating against HBV in CLD patients is not commonly implemented, often leading to delayed discovery of the patient’s immunity status.

Among vaccinated CLD patients, response rates to the hepatitis B vaccine, particularly in those with cirrhosis, are suboptimal, ranging from 16 to 88% [8,21]. This disparity in seroconversion rate seems to be primarily attributable to the presence/absence of liver cirrhosis at diagnosis. Moreover, the selected vaccine regimen—single/double dose, and undetectable Anti-HBs prior to vaccination are other independent factors [5,8,22,23].

The introduction of *Heplisav-B*, a hepatitis B vaccine with a novel adjuvant approved in the United States in 2017 and later in Europe in 2021, offers a potential alternative. Early retrospective studies suggest that it might provide increased response rates in CLD patients compared to conventional vaccines (67.5% compared to 33–45%) [24,25]. However, this response rate declined significantly in patients >50 years of age. Therefore, comprehensive data supporting its routine use in this specific patient group are still lacking. Similarly, *PreHevbrio* is a highly immunogenic 3-antigen (S/pre-S1/pre-S2) hepatitis B vaccine (3A-HBV) that recently received marketing authorization in the United States (2021) [26] the European Union, United Kingdom (2022—brand name *PreHevbri*), and Canada (2022—brand name *PreHevbrio*) for the prevention of infection caused by all known subtypes of the hepatitis B virus and the delta virus in adults 18 years and older [26]. It is a highly immunogenic vaccine, which elicits a more potent and long-lasting immune response compared to conventional vaccines [27]. Of note, both Heplisav-B and *PreHevbrio* are not yet approved for use in pregnant or breastfeeding women.

For CLD patients, particularly those with cirrhosis, the threshold for poor response is an important consideration. The definition of a poor response is generally considered anti-HBs titer less than 10 milli-international units/mL after vaccination [28]. This low response necessitates alternative strategies, such as administering a higher or double dose of the vaccine, to improve the chances of achieving adequate immunity. Our assessment is in line with other societies guidelines recommending annual to bi-annual monitoring of anti-HBs to assure adequate immunity [29] (Table 1).

Current research is focused on developing strategies to enhance immunogenicity and improve application methods. A significant development in this area is intradermal inoculation. This method, explored for its potential to be more immunogenic than traditional intramuscular injections, could offer a promising alternative for increasing vaccine efficacy, especially in patients who have shown poor responses to standard vaccine regimens [29,30].

DNA vaccines represent another innovative approach. These vaccines, which contain naked DNA (plasmids with the HBV S gene), work by expressing the hepatitis B surface antigen (HBsAg) directly in muscle cells. This method stimulates both an antibody response and cellular immunity [31,32]. The intracellular production of HBsAg may lead to a more durable and robust immune response against hepatitis B, offering a new avenue for enhancing vaccine effectiveness, particularly for those with suboptimal responses to conventional vaccines.

Furthermore, the efficacy of vaccines that combine HBsAg with various adjuvants is being actively investigated. This strategy is aimed at increasing the immunogenicity of the vaccine, making it more effective for patients who typically show poor responses to traditional hepatitis B vaccines. By combining HBsAg with different adjuvants, research is underway exploring tailored solutions to enhance protection in vulnerable patient groups [33,34].

### 3.2. Influenza

Influenza vaccination is essential for patients with CLD risk for severe complications from influenza. However, the effectiveness of the influenza vaccine in this population has not been extensively studied.

A systematic review and meta-analysis found that CLD patients, both with and without cirrhosis, do respond to influenza vaccination, but the quality of evidence was very low. The study suggested that vaccination might reduce the risk of hospital admission in patients with viral liver disease, indicating a potential benefit of vaccination in reducing hospital admissions [9]. Despite this, no significant effect against all-cause or cause-specific mortality or hospitalization was found.

Another study underscored an increasing willingness among patients with chronic kidney or liver disease (CKD/CLD) to receive influenza vaccinations, yet it reveals a concerning gap, as actual vaccination rates linger below 50%. This trend mirrors similar challenges in other vaccinations for CLD patients, where adherence to national recommendations remains a significant hurdle [9].

Recent advancements in mRNA vaccine technology, as seen with the COVID-19 vaccines, are paving the way for similar approaches in influenza vaccine development. mRNA vaccines offer several advantages, including rapid development and production, and the ability to elicit a strong immune response. Researchers are exploring lipid nanoparticle delivery systems for mRNA vaccines, which not only efficiently express the mRNA-encoded immunogen but also act as adjuvants to enhance the immune response [35,36].

Additionally, there is interest in leveraging nanotechnology for vaccine development. Nanomaterials can serve as ideal carriers for antigen delivery, acting as adjuvants and mimicking viral structures to enhance the immune response. The success of lipid nanoparticles in delivering mRNA vaccines in clinical trials for COVID-19 is encouraging for their potential application in influenza vaccines [36].

In the meantime, it is advisable for CLD patients to receive the influenza vaccine annually, as recommended by health authorities. Healthcare providers should emphasize the importance of vaccination to CLD patients and ensure they are informed about the benefits and availability of the vaccine. Additionally, healthcare systems should consider strategies to improve access and adherence to influenza vaccination among CLD patients, including patient education, reminders, and streamlined vaccination processes within primary care settings and hospitals (Table 1).

### 3.3. COVID-19

The COVID-19 pandemic has significantly shifted the landscape of vaccine knowledge and development, particularly with the introduction of mRNA vaccines. This novel type of vaccine, utilized for the first time on a large scale since their conceptual inception, represents a groundbreaking advancement in immunization technology. Studies have shown that patients with CLD, including those with compensated or decompensated liver cirrhosis, exhibit a decreased immunologic response to COVID-19 vaccines. Despite this, vaccination has been associated with a reduction in mortality in CLD patients [37,38].

In a study focusing on patients with cirrhosis following mRNA COVID-19 vaccination, it was found that these patients had impaired T-cell and antibody responses with more advanced cirrhosis associated with poorer immune responses [39]. Another study reported that three doses of the COVID-19 mRNA vaccine resulted in high antibody concentrations in immunocompromised individuals, including those with liver cirrhosis. The anticipated hybrid immunity, which is a combination of immunity induced by vaccination and natural infection, led to significantly higher antibody levels than vaccine-induced immunity alone [39,40], often reaching levels comparable to healthy individuals indicating a catch-up effect. Whether this significant seroconversion results in heightened protection against SARS-CoV-2 is a matter of debate. The rate at which protection wanes is also unknown.

A further investigation into the immune functions of cirrhotic patients’ post-vaccination revealed that the presence of myeloid-derived suppressor cells (MDSCs) was associated with impaired antigen-specific T cell reactivity. This finding suggests that MDSCs may mediate immunosuppression, leading to deficient vaccine-specific T cell responses in cirrhosis [40].

Based on our findings, we are rigorously investigating the longevity of both humoral and cellular immune responses in CLD patients following repeated COVID-19 vaccinations. Preliminary results indicate a rapid decline in the immunization’s protective effect, which will be detailed in an upcoming manuscript. Our aim is to update vaccination schedules and define protection criteria for this at-risk group. Presently, we recommend yearly vaccination, aligning with the seasonal flu shot timing. For those who have recently recovered from COVID-19, we advise waiting three months before vaccination. We do not recommend antibody titer monitoring post-vaccination (Table 1).

### 3.4. Respiratory Syncytial Virus (RSV)

The recent approval of Arexvy (GSK) and Abrysvo (Pfizer) vaccines against Respiratory Syncytial Virus (RSV) by the FDA and EMA constitutes a considerable advancement in the prophylactic arsenal of public health, particularly for subpopulations at elevated risk of severe outcomes, such as patients with CLD. The critical role of these vaccines emerges against the backdrop of evidence from systematic reviews [41], which has delineated the burden of RSV in older adults as analogous to that of influenza—as previously mentioned, a pathogen well-characterized for its potential to precipitate acute and severe respiratory pathology in this group of patients.

Arexvy contains a recombinant RSV prefusion F protein (RSVPreF3 OA) adjuvanted with AS01_E, which is known to enhance the body’s immune response to the antigen. The efficacy of Arexvy has been demonstrated to be 82.6% in the prevention of RSV-related lower respiratory tract disease and 94.1% in the prevention of severe disease manifestations among adults aged 60 and above [42]. Abrysvo, on the other hand, while also utilizing subunit technology, is distinguished by its bivalent formulation, targeting two different antigens of the RSV. It has shown an effectiveness of 89% against RSV-related pulmonary infections in older adults during the first RSV season following vaccination [43].

Both vaccines represent a significant step forward in the prevention of RSV, particularly among older adults who are at an increased risk of severe respiratory illness. The subunit technology utilized in both vaccines provides an advantage in terms of safety and efficacy, as it does not involve the introduction of live virus particles, thus reducing the risk of vaccine-induced disease, which is an important consideration for immunocompromised individuals, such as those with CLD.

Given that the clinical trials predominantly enrolled older adults, many of whom had multiple chronic conditions, it is scientifically tenable to extrapolate these benefits to individuals with CLD. However, this extrapolation warrants further empirical investigation to determine the precise efficacy and safety profile of these vaccines in the CLD cohort (Table 1). A major limitation in assessing the rate and durability of seroconversion for RSV lies in the lack of a reliable serological assay. This is primarily due to significant cross-reactivity with other viruses in the Pneumoviridae family. In contrast to SARS-CoV-2, where virtually no pre-existing immunity existed prior to 2019, a substantial portion of the population has pre-existing immunity to RSV, which is acquired during childhood. This factor necessitates a robust methodological approach to measure RSV infection prevalence in a large and diverse population sample, ensuring that the findings are statistically representative and conclusive. Currently, access to these vaccines in Sweden is limited, but projections suggest potential availability by 2024.

### 3.5. Pneumococcal Vaccine

Patients with CLD, especially older individuals, cigarette smokers, and those with chronic alcohol abuse, are at an increased risk of infections caused by Streptococcus pneumoniae (S. pneumoniae) [44]. This risk is significantly elevated in the CLD population, with a 2- to 13-fold higher likelihood of developing invasive pneumococcal disease compared to the general population, which is a factor that varies with age [45]. Furthermore, S. pneumoniae is commonly responsible for spontaneous bacterial peritonitis in cirrhotic patients [46]. Three vaccines are currently available in the USA and Europe, the pneumococcal conjugate vaccine 13 (PCV13 or Prevnar 13), pneumococcal conjugate vaccine 20 (PCV20 or Apexxnar—EU/Prevnar 20—USA), and the pneumococcal polysaccharide vaccine (PPSV23 or Pneumovax 23). However, evidence about the effectiveness and lasting immunity of those vaccines in immunocompromised groups, including those with CLD and chronic kidney disease, is sparse and inconclusive. Studies in both demographics have shown mixed results. For instance, cirrhotic patients, particularly those evaluated for LT, displayed varying immunoglobulin responses compared to controls with a rapid decline in antibody levels post-transplantation [47]. Similarly, research in chronic kidney disease patients revealed no significant protective effects of PPSV23 against all-cause death, pneumonia, acute myocardial infarction, or cerebrovascular events [48]. While the study did not focus on CLD, some findings might be extrapolated to the CLD population.

Given this limited and somewhat discouraging evidence, our recommendation is to prioritize vaccination for cirrhotic patients with a history of prior decompensation as well as those on the LT waiting list.

In LT patients, both vaccines can be used. The vaccination process should ideally commence before LT, or if not feasible, no earlier than 6 months post-transplantation. For previously unvaccinated patients, a single dose of Apexxnar is recommended. Patients who have received Pneumovax in the past should be administered one dose of Apexxnar but only after at least one year has passed since the last Pneumovax dose. In cases where patients have previously been vaccinated with both PCV13 and PPSV23, a dose of PPSV23 or PCV20 is advised to be given 5-6 years following their most recent vaccination dose (Table 1).

### 3.6. Varicella Zoster and Herpes Zoster

Varicella zoster virus (VZV) infection and its reactivation as herpes zoster (shingles) are of particular concern in patients on immunosuppression with mycophenolate mofetil and post-LT [49]. For primary varicella zoster infection, evidence regarding vaccine efficacy in cirrhosis patients is limited but suggests that the response to vaccination can be variable. Two vaccines are currently available, Varilrix and Varivax, and both are live-attenuated vaccines. A study on pediatric LT patients showed that booster vaccinations could achieve long-term VZV immunity comparable to healthy controls, although the response to vaccination was weaker and wanes quickly compared to healthy children [50]. Another ambitious study in LT recipients indicated that the recombinant subunit herpes zoster vaccine (Shingrix) was safe and elicited significant humoral and cellular responses, suggesting its potential as a preventive strategy against primary varicella [51].

In the context of herpes zoster, two vaccines are available: the live-attenuated zoster vaccine (Zostavax) and the recombinant zoster vaccine (Shingrix). The latter, being nonreplicating, is particularly suitable for cirrhotic patients. It has shown greater efficacy compared to LZV and is recommended for herpes zoster prevention in this population [52].

Our recommendation is to screen and immunize (when resources are available) all patients with CLD against primary varicella zoster infection. For herpes zoster, we recommend vaccinating all patients with cirrhosis Child–Pugh B and C and those on the LT waiting list. In Sweden, patients in this group must pay out of pocket for this vaccination.

In the case of LT, for patients lacking immunity to VZV, the live-attenuated virus vaccines Varilrix and Varivax are recommended. These vaccines should be administered in two doses, spaced (4)-6 weeks apart, and ideally, the vaccination course should be completed at least (4)-6 weeks before LT. If LT occurs within one month after vaccination, acyclovir treatment is advised. Approximately 6 weeks post-vaccination, antibody levels against VZV should be checked. Current guidelines advise against vaccinating already transplanted patients who lack antibodies against VZV. However, family members and hospital staff without immunity to VZV should be vaccinated. For patients over 50 years old who are immune to chickenpox, the non-live Shingrix vaccine for shingles can be considered. It is administered in two doses with an interval of 2(-6) months. The vaccination is preferably completed before LT or, if not feasible, at least 18 months post-LT (Table 1).

On the research and development front, the breakthroughs with mRNA vaccines for COVID-19 have opened new avenues in vaccine research. These vaccines use messenger RNA to instruct cells to produce proteins that mimic viral antigens, thereby stimulating an immune response. Research efforts are now being directed toward applying this technology to herpes zoster with clinical trials aiming to evaluate their efficacy and safety [49]. For instance, in a nonhuman primate model, an mRNA-lipid vaccine expressing VZV glycoprotein gE induced gE-specific antibody and CD4^+^ T-cell responses comparable to RZV and superior to those induced by ZVL [53].

### 3.7. Tuberculosis (TB)

In cirrhotic patients, the Bacillus Calmette–Guérin (BCG) vaccine’s efficacy is compromised due to the vaccine’s ability to evade phagosome maturation and autophagy, and its reduction in MHC-II expression on antigen-presenting cells, which are crucial for T-cell activation. Cirrhosis further impairs these immune processes, particularly affecting the phagocytic function of Kupffer cells and leading to weakened antigen processing and presentation. Additionally, cirrhosis-associated alterations in lymphocyte function and the cytokine environment further diminish T-cell responses essential for effective defense against TB. This complex interplay between BCG’s immune evasion mechanisms and the immunological impairments associated with cirrhosis challenges the vaccine’s effectiveness in this patient group [54]. Despite this, studies that specifically evaluated the vaccine’s response in cirrhotic patients, via antibody levels or Interferon-Gamma Release Assays (IGRAs), are scarce. BCG is widely recognized for its protective role against TB in children, but its effectiveness in adults with conditions like CLD or cirrhosis remains uncertain. The immune response to BCG vaccination can be measured using the tuberculin skin test (TST) or IGRA, which detect immune reactivity to mycobacterium tuberculosis antigens. Yet, these tests cannot distinguish between responses to vaccination and actual tuberculosis exposure [55]. Presently, no serological test exclusively assesses the immune response to the BCG vaccine.

Concerns about the potential activation of tuberculosis following BCG vaccination in immunocompromised individuals require careful consideration of the vaccine’s safety and effectiveness in these patients [56]. Emerging research into modified BCG vaccines, such as the recombinant BCG expressing LTAK63 (rBCG-LTAK63), suggests they could offer safer and more efficacious alternatives for immunocompromised individuals [54,57]. Due to the insufficient data on BCG vaccine efficacy in CLD patients and the associated risks, our recommendation is to refrain from administering the BCG vaccine to this group in adult age (Table 1).

### 3.8. Measles, Morbilli and Rubella (MMR)

In the population with CLD, standard immunity from childhood MMR vaccinations is presumed to some extent. Nonetheless, studies have shown that measles immunity is not lifelong with antibody titers potentially declining below the protective level of 200 mIU/mL after approximately 15 years [58]. The altered immune landscapes in patients with CLD may further diminish the longevity and effectiveness of the MMR vaccine.

In Sweden, patients with CLD who are on concomitant treatment with immunosuppressive drugs are managed carefully regarding live vaccines such as MMR. The cessation of immunosuppressive therapy three months prior to vaccination is recommended to improve immunogenicity, which is followed by a serological assessment after three months to gauge the response before the restarting immunosuppression. This protocol serves to optimize immunological response and patient safety. Given the scarcity of systematic surveillance for immunization status in CLD patients of different etiologies, our recommendation is to administer a booster dose of the MMR vaccine soon after diagnosis to bolster protective immunity in this vulnerable group (Table 1).

### 3.9. Tetanus, Diphtheria and Pertussis

The efficacy and safety of tetanus, diphtheria, and pertussis vaccinations in patients with CLD are strongly supported by scientific evidence, leading to robust guidelines for their administration. The tetanus vaccine (Boostrix), commonly included in the DTaP (Diphtheria, Tetanus, and acellular Pertussis) is recommended for adults with cirrhosis every 10 years [59,60]. In cases of injury, a booster may be required sooner if more than 5 years have elapsed since the last dose. If the patient is planning a trip to a country where polio is present, we recommend administering a DTaP combination (Boostrix Polio). Similar recommendations apply for diphtheria and pertussis (Table 1).

### 3.10. Human Papilloma Virus (HPV)

There is an established causal link between human papillomaviruses (HPVs) and certain cancers, including cervical cancer. The HPV vaccine has been in clinical use for over a decade with varying administration policies globally. Its use typically does not extend beyond early adulthood in women. Currently, there is a notable lack of specific research on the immunogenicity or efficacy of the HPV vaccine in CLD and post-LT patients. As a result, much of the existing data are extrapolated from studies on similar population groups [61]. Nevertheless, the vaccine is recommended for organ transplant recipients due to their increased risk of HPV-related cancers [62]. For CLD patients without LT, vaccination recommendations should be guided by local expertise and practice patterns (Table 1).

### 3.11. Tick-Borne Encephalitis (TBE)

In areas with a significant presence of tick-borne encephalitis (TBE), such as Sweden, the TBE vaccine is highly recommended, especially for those engaging in activities in tick-infested environments. The standard vaccination schedule involves three doses at months 0, 1, and 12 with an additional priming dose at month 3 for individuals aged 60 and above [63]. Boosters are advised every 5 years before age 60 and every 3 years thereafter beyond [64]. For CLD patients, the efficacy of this schedule may vary. Therefore, it is advised to tailor the immunization approach based on local guidelines and individual health considerations [65] (Table 1).

## 4. Discussion and Future Directions

In the context of CLD, with or without cirrhosis, there is a heightened risk for patients contracting severe illnesses from viral or bacterial sources. These preventable infections often lead to hospital stays and subsequent readmissions, significantly straining healthcare resources. For instance, one meta-analysis reported that in patients with cirrhosis, infections increase mortality 4-fold, where 30% of patients die within 1 month after infection and another 30% die by 1 year [66]. Implementing vaccinations early in the disease’s progression can mitigate these risks, providing a cost-effective and often a reliable method to minimize morbidity in this susceptible group. Although vaccinations against hepatitis A and B are recommended for patients with CLD shortly after diagnosis, it is observed that a significant number of patients do not undergo routine screening and vaccination for these diseases particularly when the etiology of the liver disease is not viral. In one report, the vaccination rate to hepatitis A and B was as low as 16% [67]. The predominant reason for this lack of compliance was identified as the absence of discussion by the physician, where the risk/benefit analysis of vaccination is not addressed during clinical appointments—in 31% to 78% of cases, which is followed by loss to follow-up (35%). Moreover, factors such as etiology of CLD, higher Child–Pugh score, older age, and lower education level are also independently associated with lower vaccination rates [68,69].

Acknowledging the challenges in hepatitis vaccination uptake underscores the broader issue of vaccine administration in CLD patients, including those for Tdap, seasonal COVID-19, influenza, and pneumococcal diseases. The efficacy of the simultaneous administration of these vaccines after a CLD diagnosis remains undetermined in this population due to the heterogeneity of published reports [70]. However, given the lack of concrete data, it seems prudent to administer these vaccines concurrently to improve adherence. Particular attention should be given to CLD patients with respiratory conditions, prioritizing pneumococcal and influenza vaccines. Furthermore, the co-administration of COVID and influenza vaccines, injected at separate sites, aligns with National Health Authority guidelines, and is deemed safe.

Despite strong endorsements from expert panels and health authorities, vaccination rates in CLD patients are disappointingly low. Several factors contribute to this, including limited healthcare access, insufficient awareness about vaccination guidelines among healthcare providers and patients, vaccine cost issues, inadequate financial support for healthcare providers, challenges in completing vaccine courses, and prevalent vaccine misinformation. Several strategies have been suggested to enhance immunization rates, such as incentives for completing vaccination series, integrating vaccine records into electronic medical systems as a quality measure for primary care, and using digital communication tools for vaccination reminders. Other physician-level interventions include regular vaccination status assessments during clinic visits with electronic health record alerts, reducing patient out-of-pocket costs, and arranging home vaccination visits. Additionally, the integration of local pharmacies with vaccination clinics, which facilitates a more streamlined vaccination process, could play a significant role in identifying and vaccinating at-risk patients in coordination with primary care doctors. Overcoming these barriers to improve vaccination coverage in CLD patients demands a comprehensive, multidisciplinary approach.

Vaccine hesitancy has become increasingly prevalent in recent years, which is driven by a complex interplay of factors. Misinformation and disinformation spread through social media and other digital platforms have played a significant role, sowing doubts about vaccine safety and efficacy [71]. Additionally, a lack of trust in pharmaceutical companies and government health agencies has further fueled skepticism. Historical instances of unethical medical practices have also contributed to mistrust, particularly among marginalized communities where CLD is more prevalent. The consequences of vaccine hesitancy are profound, including the resurgence of previously controlled diseases, outbreaks of new infectious diseases, and increased morbidity and mortality. These outcomes not only threaten public health but also impose significant economic burdens due to increased healthcare costs and lost productivity [72,73]. Addressing vaccine hesitancy requires a multifaceted approach, including improving public education on vaccines, enhancing transparency and accountability in the pharmaceutical industry, and building trust through community engagement and participation.

The field of vaccine technology is rapidly evolving with mRNA vaccines leading the charge, which are followed by DNA vaccines, nanoparticle-based vaccines, viral vector vaccines, microneedle patch vaccines, and intranasal vaccines. While these innovations are promising, their integration into clinical practice is still in progress. Bridging this gap is crucial, particularly for immunocompromised patients who stand to benefit from more effective, personalized vaccination methods. The transition of these technologies from research to clinical use requires extensive clinical trials, regulatory clearances, and assessments of real-world effectiveness. While the landscape of CLD vaccination currently depends on traditional vaccines, these emerging technologies hold the promise of revolutionizing immunization strategies for vulnerable groups in the future.

## Figures and Tables

**Table 1:** Vaccine recommendations for patients with chronic liver disease, including those at different stages of cirrhosis (Child–Pugh A, B, C) and post-liver transplant (post-LT).

Disease/Vaccine	CP A	CP B	CP C	Post-LT *	Notes
**Hep A and B**	Yes	Yes	Yes	Yes	Booster recommended if anti-HBs < 10 IU/L. Anti-HAV measurement not recommended.
**Influenza**	Yes	Yes	Yes	Yes	Annual vaccination essential. Vaccination for same household contact is also recommended.
**COVID-19**	Yes	Yes	Yes	Yes	Annual vaccination essential. Vaccination for same household contact is also recommended.
**RSV**	Yes	Yes	Yes	Yes	Annual vaccination recommended >60 years old. Not to be combined with other vaccines.
**Pneumococcal disease**	Yes	Yes	Yes	Yes	Prioritize for those with prior decompensation, on LT waiting list and post-LT.
**Varicella zoster**	Yes	Yes	Yes	Yes	Screen and immunize against primary VZV infection; preferably before LT.
**Herpes zoster**	No	Yes	Yes	Yes	Prioritize for patients over 50 years.
**TB**	No	No	No	No	BCG vaccine efficacy in CLD patients uncertain; concerns about potential activation.
**MMR**	Yes	Yes	Yes	Yes	Booster dose recommended soon after CLD diagnosis; acquired immunity is not lifelong.
**DTaP**	Yes	Yes	Yes	Yes	Recommended every 10 years or booster if 5 years elapsed since last dose; Boostrix Polio for travel to polio-endemic areas.
**HPV**	Yes	Yes	Yes	Yes	Recommended post-LT; 3-dose schedule for advanced cirrhosis.
**TBE**	Yes	Yes	Yes	Yes	Highly recommended in endemic areas.

The recommendations are color-coded to indicate their strength. Green—strong recommendation for vaccination; Yellow—weak recommendation, may depend on individual circumstances; Red—not recommended, or recommended with significant reservations. CLD—chronic liver disease; CP—Child–Pugh; LT—liver transplant; Hep A—hepatitis A; Hep B—hepatitis B; RSV—respiratory syncytial virus; VZV—varicella zoster virus; TB—tuberculosis; MMR—measles, mumps, and rubella; DTaP—Diphtheria, Tetanus, and Pertussis; HPV—human papillomavirus; TBE—tick-borne encephalitis. * Exercise caution in patients on immunosuppressant if live-attenuated vaccines are planned.

## Abbreviations

CLD—chronic liver disease, COVID-19—Coronavirus Disease 2019, CAID—cirrhosis-associated immune dysfunction, HAV—hepatitis A virus, HBV—hepatitis B virus, AASLD—American Association for the Study of Liver Diseases, EASL—European Association for the Study of the Liver, mRNA—Messenger Ribonucleic Acid, RSV—Respiratory Syncytial Virus, FDA—Food and Drug Administration, EMA—European Medicines Agency, PCV13—pneumococcal conjugate vaccine 13, PCV20—pneumococcal conjugate vaccine 20, PPSV23—pneumococcal polysaccharide vaccine 23, VZV—varicella zoster virus, LT—liver transplantation, MMR—measles, mumps, and rubella, TBE—tick-borne encephalitis, HPV—human papillomavirus, BCG—Bacillus Calmette–Guérin, IGRA—Interferon-Gamma Release Assays, TST—tuberculin skin test, DTaP—Diphtheria, Tetanus, and Pertussis, *S. pneumoniae*—Streptococcus pneumoniae, ACIP—Advisory Committee on Immunization Practices.
